# Principal Component Analysis and Risk Factors for Acute Mountain Sickness upon Acute Exposure at 3700 m

**DOI:** 10.1371/journal.pone.0142375

**Published:** 2015-11-10

**Authors:** Shi-Zhu Bian, Jun Jin, Ji-Hang Zhang, Qian-Ning Li, Jie Yu, Shi-Yong Yu, Jian-Fei Chen, Xue-Jun Yu, Jun Qin, Lan Huang

**Affiliations:** 1 Institute of Cardiovascular Diseases of PLA; Xinqiao Hospital, Third Military Medical University, Chongqing, China; 2 Department of Cardiology, Xinqiao Hospital, Third Military Medical University, Chongqing, China; 3 Department of Neurology, Xinqiao Hospital, Third Military Medical University, Chongqing, China; Boston University School of Medicine, UNITED STATES

## Abstract

**Objective:**

We aimed to describe the heterogeneity in the clinical presentation of acute mountain sickness (AMS) and to identify its primary risk factors.

**Methods:**

The participants (n = 163) received case report form questionnaires, and their heart rate (HR), oxygen saturation (SpO_2_), echocardiographic and transcranial Doppler variables, ability to perform mental and physical work, mood and psychological factors were assessed within 18 to 22 hours after arriving at 3700 m from sea level (500 m) by plane. First, we examined the differences in all variables between the AMS-positive and the AMS-negative groups. Second, an adjusted regression analysis was performed after correlation and principal component analyses.

**Results:**

The AMS patients had a higher diastolic vertebral artery velocity (V_d_; p = 0.018), a higher HR (p = 0.006) and a lower SpO_2_. The AMS subjects also experienced poorer sleep quality, as quantified using the Athens Insomnia Scale (AIS). Moreover, the AMS population exhibited more negative mood states, including anxiety, depression, hostility, fatigue and confusion. Five principal components focused on diverse aspects were also found to be significant. Additionally, more advanced age (p = 0.007), a higher HR (p = 0.034), a higher V_d_ (p = 0.014), a higher AIS score (p = 0.030), a decreased pursuit aiming capacity (p = 0.035) and decreased vigor (p = 0.015) were risk factors for AMS.

**Conclusions:**

Mood states play critical roles in the development of AMS. Furthermore, an elevated HR and V_d_, advanced age, elevated AIS sores, insufficient vigor and decreased mental work capacity are independent risk factors for AMS.

## Introduction

Acute mountain sickness (AMS) occurs in individuals who ascend to altitudes of 2500 m or higher [1–3], and it is diagnosed using the Lake Louise Score (LLS), a self-scoring system [2–4]. AMS is characterized by headache, dizziness, difficulty sleeping, fatigue and gastrointestinal symptoms. This condition has been recognized as a non-fatal syndrome that limits one’s daily life and work at high altitudes, especially for newcomers from sea level. Thus, critical attention should be focused on the characteristics of AMS upon acute high-altitude exposure.

Though it has been researched for hundreds of years, the underlying mechanisms of AMS are not fully understood. These mechanisms involve alterations in the cardiovascular system, including changes in the heart rate (HR), cardiac output (CO), respiratory responses and cerebral blood flow (CBF) [5, 6]. However, the role of other systemic hemodynamic parameters in AMS has not been studied [7–11]. AMS and high-altitude cerebral edema share certain pathophysiological mechanisms and clinical characteristics, including headaches [12]. Our previous study indicated that AMS was related to posterior cerebral circulation (velocities in the vertebral artery), rather than the anterior cerebral circulation (velocities in the middle cerebral artery) [6]. Thus, cerebral hemodynamics [5, 6], cognitive functions or the mental work capacity required by the subject and his or her emotional state may also be risk factors for AMS.

Currently, AMS is diagnosed based on subjective, self-reported symptoms. Thus, no highly effective, accurate objective criteria are used to evaluate AMS. Although many studies have investigated certain aspects of AMS, the psychological and emotional manifestations of this disorder and the increased mental effort required in subjects who are exposed to high altitudes have not received attention. Here, we postulate that AMS may be characterized by specific psychological and physiological patterns. Therefore, we integrated demographic, psychological, physiological, mental and emotional data to describe the heterogeneity in the clinical presentations of individuals acutely exposed to high altitude to facilitate our understanding of the disease process, to provide insight into the specific phenotypes associated with the course of the disease and to identify the primary risk factors for AMS.

## Methods

### Participants and procedures

#### Participants

A total of 163 subjects participated in the study. The inclusion criteria included being healthy, male and between 18 and 60 years of age; residing at sea level; and being a newcomer to high altitude, without past high-altitude exposure. The exclusion criteria including the follows: people who have hypertension, arrhythmia, myocarditis and other cardiovascular diseases, primary headache, acute mountain sickness histories, a cold, pneumonia, pulmonary tuberculosis and other respiratory diseases, disorders of the liver or kidneys, malignant tumors and neuropsychosis.

The study was thoroughly explained to all of the subjects who agreed to participate, and all of the subjects signed informed consent forms before they were examined. This study was reviewed and approved by the Ethics Committee of Xinqiao Hospital of Third Military Medical University.

#### Procedures

The participants were recruited in June 2012, and field trials were performed within 18 to 24 hours after the participants’ arrival at 3700 m from sea level (Chengdu in Sichuan province, 500 m) via a two-hour plane ride. Each field trial was performed in the morning after an overnight fast, and coffee, tea, and other caffeine-containing drinks as well as alcohol were avoided before the examinations to prevent these factors from affecting the examinations.

Structured case report form questionnaires were used to record demographic data (*i*.*e*., age, body mass index (BMI), smoking, alcohol consumption, educational background and occupation), symptoms of AMS and physical labor intensity. AMS was diagnosed based on the LLS (Document A in S1 File).

The subjects’ systolic blood pressure (SBP), diastolic blood pressure (DBP), heart rate (HR) and oxygen saturation (SpO_2_) were measured with a sphygmomanometer (HEM-6200, OMRON, China) and a pulse oximeter (NONIN-9550, Nonin Onyx, USA) placed on the right wrist, which was raised close to the position of the heart after the subjects had sat at rest for 30 min. Transcranial Doppler (TCD) sonography examinations were performed by the same technician, who used an ultrasonography system with a 2 Hz probe (EME TC2021-III, NICOLET, USA). In particular, after a rest of 30 min, the subjects were placed in the supine position and then in the prone position to undergo TCD examinations of the different cerebral arteries. The cerebral posterior circulation (*i*.*e*., velocities in the vertebral artery) in the subjects was recorded. Each subject received an echocardiographic examination (ultrasonography system, CX50, Philips, USA) involving measurements of the end-diastolic internal diameters of the left atrium (LA), left ventricle (LV), right atrium (RA), right ventricle (RV) and pulmonary artery (PA) as well as measurements of the stroke volume (SV) and ejection fraction (EF).

Psychological questionnaires were also administered using the Self-Rating Anxiety Scale (SAS; Document B in S1 File). The profile of mood states (POMS), including the degrees of anxiety, depression, hostility, vigor, fatigue and confusion experienced by the subjects (Document C in S1 File), was assessed as well. Moreover, sleep measurements via the Epworth Sleepiness Scale (ESS) and Athens Insomnia Scale (AIS) were employed to assess the subjects’ sleeping patterns (Documents D-E in S1 File).

The cognitive function or mental effort required by each subject to perform certain tasks was evaluated using five items. More specifically, simple reaction time, attention span, and digit span were measured using subtests from the Wechsler adult intelligence scale (WAIS) and the Wechsler memory scale (WMS), and digit symbols were measured using the WAIS subtest. Additionally, pursuit aiming capacity was also measured (Document F in S1 File).

The Fatigue Self-Assessment Scale (FSAS) was applied to quantify the subjects’ fatigue (Document G in S1 File). The physical working capacity of each subject was also calculated using the PWC170 (*i*.*e*., the physical working capacity when the HR is 170 beats/min) and was tested via specific exercises (Document H in S1 File).

### Statistical analysis

Normally distributed variables (*i*.*e*., age, BMI, HR, SBP, DBP, SpO_2_, LA, LV, RA, RV, PA, SV, CO, EF and PWC170) were expressed as the mean ± standard deviation (SD). Non-normally distributed variables (*i*.*e*., ESS score, AIS score, SAS score, tension anxiety, depression, hostility, vigor, fatigue, confusion, simple reaction time, attention span, digit symbols, digit span, pursuit aiming score and FSAS score) were expressed as the median (interquartile range). Differences between the AMS-positive (AMS+) and the AMS-negative (AMS-) groups were examined using independent-samples t tests or the Mann-Whitney U test, as appropriate. Relationships between the AMS score and all of the aforementioned parameters were also analyzed using Spearman’s correlation.

**Fig 1:**
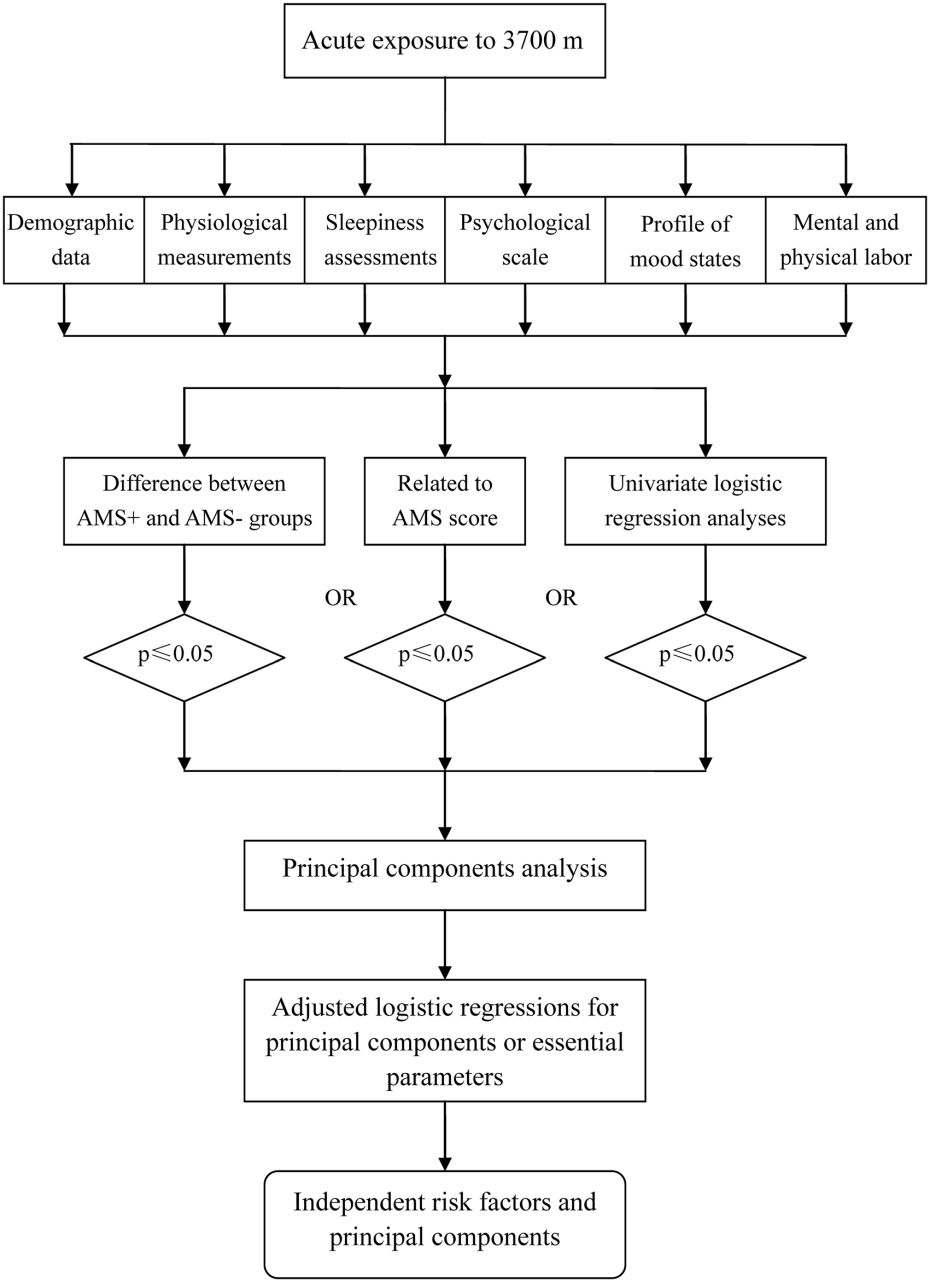
Flow diagram of the statistical analyses. An adjusted logistic regression was performed to identify independent risk factors for AMS (i.e., factors for which the p value was less than 0.05 in univariate analyses and the correlation coefficient was greater than 0.5 in principal component analysis).

Principal component analysis was performed to identify the principal components and the phenotypes associated with AMS. Principal components with an eigenvalue >1 were considered significant. Finally, the original data set was transformed using the eigenvectors as weighting coefficients to obtain principal component scores. An adjusted logistic regression was performed to identify independent risk factors for AMS (Fig 1).

Statisticians from the Third Military Medical University were consulted on all statistical methods and results.

## Results

Data were excluded if the subjects’ demographic information and other items were incomplete. Ultimately, 150 valid case report forms and examinations were collected.

**Fig 2:**
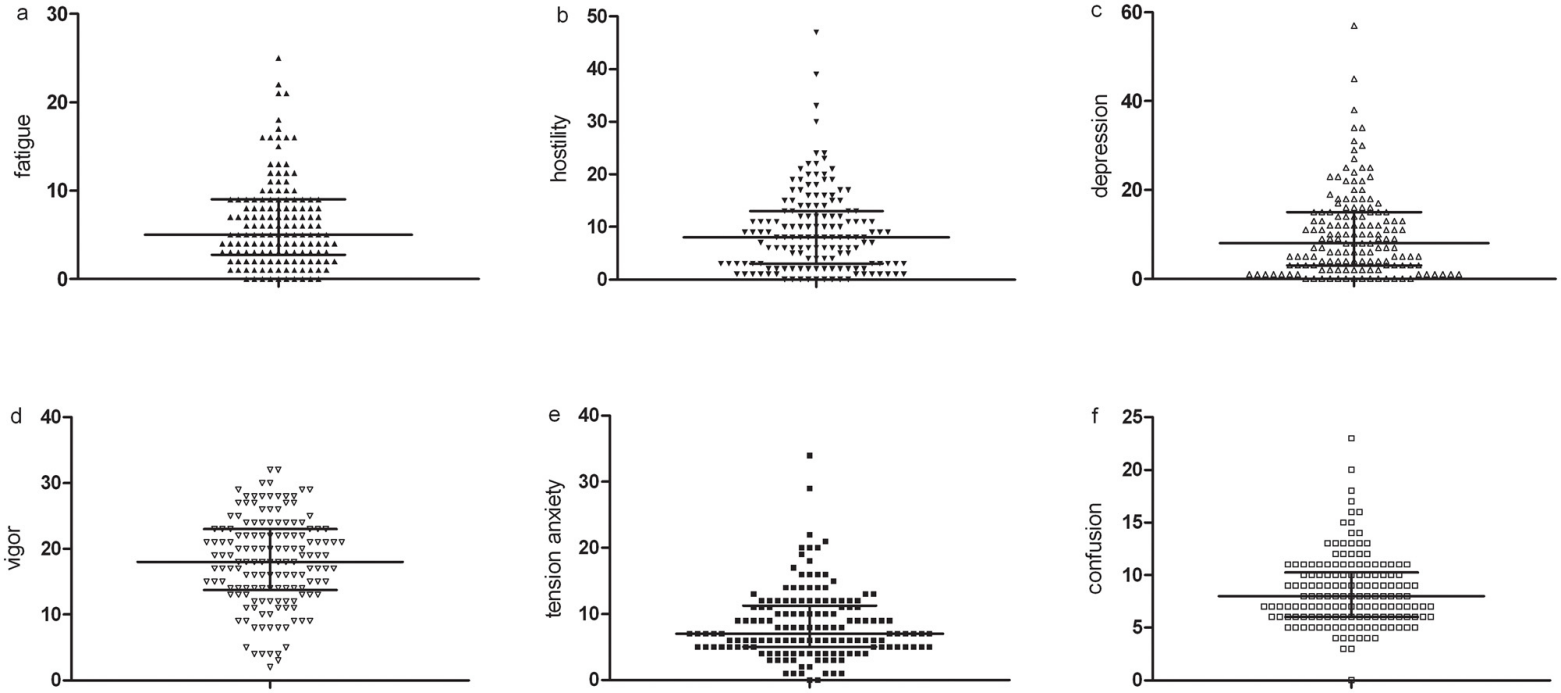
Mood states of the 150 subjects.

**Fig 3:**
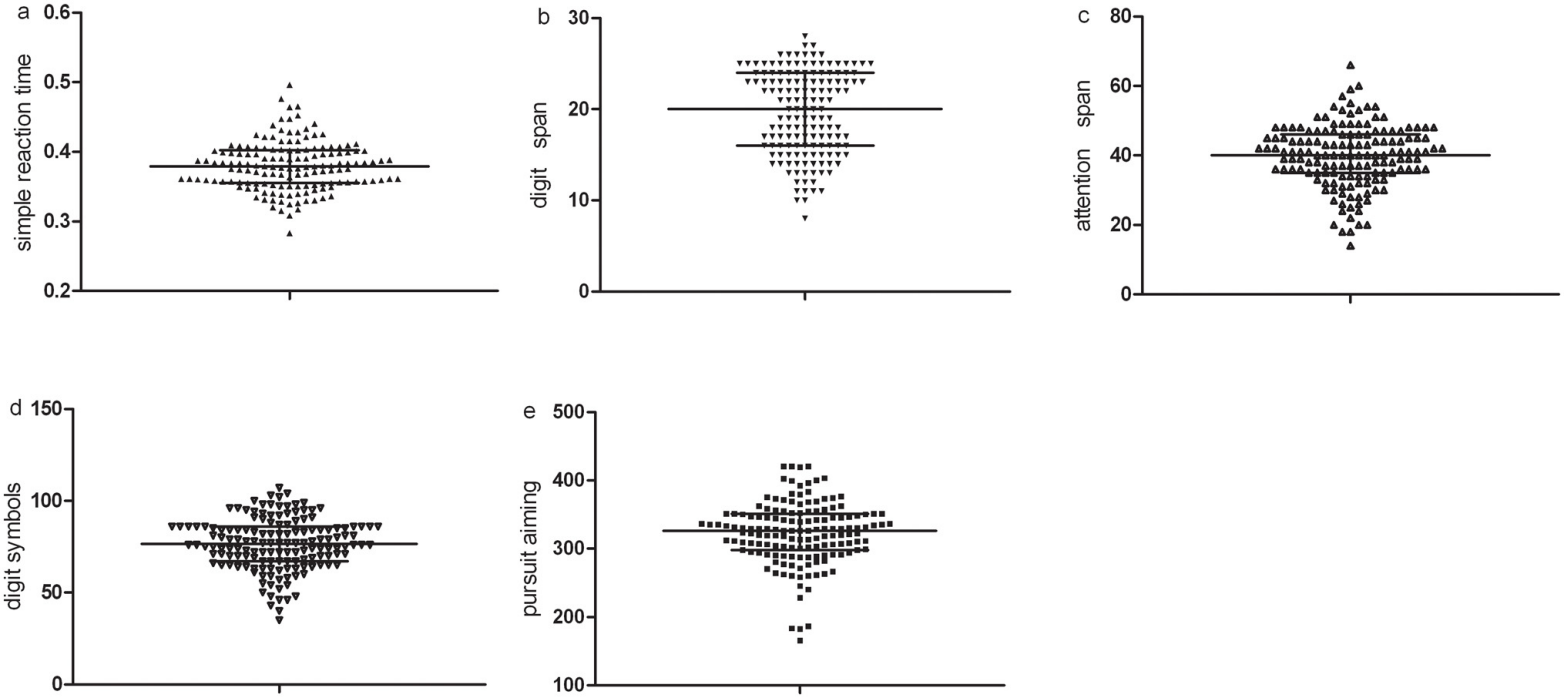
Distributions of the cognitive functions.

The mean age and BMI of the subjects were 22.17±3.33 years and 21.57±2.06 kg/m^2^, respectively, and 24.7% and 64.0% of participants smoked and drank alcohol regularly, respectively. In total, 74% of the participants were Han Chinese. Upon acute exposure to an altitude of 3700 m, 56% experienced AMS. The descriptive data regarding mood states and mental work capabilities are included in Figs 2 and 3.

Regarding the demographic data, only age and BMI were significantly higher in the AMS+ group than in the AMS- group. The patients who experienced AMS also had a significantly higher HR (p = 0.006) and diastolic vertebral artery velocity (V_d_; 25.96±3.61 *vs*. 24.47±4.01 cm/s, p = 0.018). In the sleepiness assessments, the AIS score of the AMS patients was significantly different from that of the subjects who did not experience AMS (p<0.001). In the psychological assessments, AMS+ individuals had a significantly greater degree of anxiety, as indicated by higher SAS scores (25.00 (6.00) *vs*. 22.00 (3.25), p<0.001). The POMS was also significantly different between the two groups. More specifically, anxiety (p<0.001), depression (11.00 (13.75) *vs*. 5.00 (9.25), p<0.001), hostility (10.00 (11.75) *vs*. 6.00 (7.25), p<0.001), vigor (17.00 (7.00) *vs*. 21.00 (12.00), p = 0.002), fatigue (7.00 (5.00) *vs*. 3.00 (4.25), p<0.001) and confusion (9.00 (4.00) *vs*. 7.00 (4.00), p<0.001) differed significantly between the two groups. Additionally, the variable related to the pursuit aiming capacity had a lower value in the AMS+ subjects than in the AMS- subjects (320.00 (51.25) *vs*. 332.50 (56.50), p = 0.029). Finally, the FSAS score was higher in the AMS+ group, whereas the PWC170 was similar between the two groups of subjects (Table 1).

**Table 1:** Differences in each variable between the AMS+ and the AMS- groups.

		AMS +(n = 84)	AMS-(n = 66)	p value
Demographic data				
	age	22.77±3.83	21.39±2.38	0.011*
	BMI	21.90±2.24	21.17±1.73	0.031*
	PLI	55 (65.5%)	50 (75.8%)	0.173
	smoking	21 (25.0%)	16 (24.2%)	0.915
	drinking	55 (65.5%)	41 (62.1%)	0.671
Physiological measurements				
	SBP	115.14±11.41	112.80±9.97	0.190
	DBP	75.43±9.21	73.64±10.26	0.263
	HR	86.02±12.65	80.61±10.86	0.006**
	SpO_2_	88.43±2.86	89.11±2.50	0.131
	LA	29.94±1.65	29.91±1.72	0.910
	LV	46.01±1.87	46.38±2.38	0.292
	RA	34.25±2.017	34.42±1.86	0.588
	RV	33.68±2.303	34.32±2.33	0.095
	PA	20.10±1.66	19.77±1.05	0.170
	EF	67.04±4.28	66.64±3.96	0.559
	SV	67.08±8.70	67.91±8.14	0.555
	V_m_	36.01±4.67	35.30±5.37	0.381
	V_s_	51.32±7.15	51.16±6.73	0.892
	V_d_	25.96±3.61	24.47±4.01	0.018*
	AI	0 (21.76)	-4.54 (19.02)	0.190
Sleepiness				
	ESS	12.00 (3.00)	12.00 (3.00)	0.081
	AIS	13.50 (5.00)	10.00 (3.25)	<0.001**
Psychological scale				
	SAS	25.00 (6.00)	22.00 (3.25)	<0.001**
POMS				
	tension anxiety	9.00 (3.00)	6.00 (4.00)	<0.001**
	depression	11.00 (13.75)	5.00 (9.25)	<0.001**
	hostility	10.00 (11.75)	6.00 (7.25)	<0.001**
	vigor	17.00 (7.00)	21.00 (12.00)	0.002**
	fatigue	7.00 (5.00)	3.00 (4.25)	<0.001**
	confusion	9.00 (4.00)	7.00 (4.00)	<0.001**
Mental work capacity				
	simple reaction time	0.381 (0.042)	0.372 (0.053)	0.186
	attention span	40.00 (11.75)	41.00 (12.00)	0.235
	digit symbols	76.50 (19.75)	76.50 (17.50)	0.862
	digit span	19.50 (8.75)	21.00 (7.25)	0.893
	pursuit aiming	320.00 (51.25)	332.50 (56.50)	0.029*
Physical work capacity				
	PWC170	1512.3±278.5	1522.6±319.3	0.832
	FSAS	39.00 (15.25)	34.50 (8.00)	0.001**

Age: years; BMI: kg/m^2^; smoking and drinking: %; SBP and DBP: mmHg; SpO2: %; HR: beats/min; LA, LV, RA, RV and PA: mm; SV: ml/min; EF: %; V_s_, V_d_ and V_m_: cm/s; PWC170: kg∙m/min.

*: p value is 0.05 or less;

**: p value is 0.01 or less.

The correlation analyses revealed that the subjects’ age, BMI, HR (r = 0.276, p = 0.001), SpO_2_ (r = -0.185, p = 0.024), PA (r = 0.170, p = 0.038), ESS score (r = 0.206, p = 0.011), AIS score (r = 0.638, p<0.001), SAS score (r = 0.492, p<0.001), anxiety (r = 0.437, p<0.001), depression (r = 0.392, p<0.001), hostility (r = 0.390, p<0.001), vigor (r = -0.240, p = 0.003), fatigue (r = 0.488, p<0.001), confusion (r = 0.364, p<0.001) and FSAS score (r = 0.378, p<0.001) were significantly associated with the AMS score (Table 2).

**Table 2:** Relationships between the AMS score and all of the parameters.

		r (with AMS score)	p value
Demographic data			
	age	0.189	0.021*
	BMI	0.181	0.026*
Physiological measurements			
	SBP	0.116	0.157
	DBP	0.155	0.058
	HR	0.276**	0.001**
	SpO_2_	-0.185*	0.024*
	LA	0.077	0.349
	LV	-0.042	0.612
	RA	-0.029	0.728
	RV	-0.114	0.165
	PA	0.170*	0.038*
	SV	-0.033	0.692
	EF	-0.016	0.848
	V_m_	0.048	0.560
	V_s_	-0.030	0.718
	V_d_	0.145	0.077
	AI	0.070	0.394
Sleepiness			
	ESS	0.206	0.011*
	AIS	0.638	<0.001**
Psychological scale			
	SAS	0.492	<0.001**
POMS			
	tension anxiety	0.437	<0.001**
	depression	0.392	<0.001**
	hostility	0.390	<0.001**
	vigor	-0.240	0.003**
	fatigue	0.488	<0.001**
	confusion	0.364	<0.001**
Mental work capacity			
	simple reaction time	0.030	0.718
	attention span	-0.026	0.749
	digit symbols	0.078	0.341
	digit span	0.014	0.866
	pursuit aiming	-0.124	0.131
Physical work capacity			
	PWC170	-0.001	0.990
	FSAS	0.378	<0.001**

*: p value is 0.05 or less;

**: p value is 0.01 or less.

The univariate logistic regression analyses revealed that the subjects’ age, BMI, HR, V_d_, AIS score, SAS score, anxiety, depression, hostility, vigor, fatigue, confusion, pursuit aiming and FSAS score were risk factors for AMS (*i*.*e*., all of these variables had associated p values that were less than 0.05; Table 3).

**Table 3:** Logistic regression for each variable.

Risk factor		β coefficient	Odds ratio	(95% CI)		p value
				Lower	Upper	
Demographic data						
	age	0.138	1.148	1.028	1.282	0.014*
	BMI	0.186	1.204	1.014	1.429	0.034*
	PLI	-0.499	0.607	0.295	1.248	0.174
	smoking	-0.147	0.864	0.533	1.399	0.551
	drinking	0.023	1.023	0.721	1.450	0.899
Physiological measurements						
	SBP	0.021	1.021	0.990	1.053	0.191
	DBP	0.019	1.020	0.986	1.055	0.262
	HR	0.040	1.040	1.010	1.071	0.008**
	SpO_2_	-0.094	0.910	0.806	1.029	0.132
	LA	0.011	1.011	0.834	1.227	0.909
	LV	-0.084	0.920	0.787	1.074	0.291
	RA	-0.046	0.955	0.809	1.128	0.586
	RV	-0.120	0.887	0.770	1.022	0.096
	PA	0.177	1.194	0.923	1.544	0.177
	EF	0.024	1.024	0.946	1.108	0.557
	SV	-0.012	0.988	0.951	1.027	0.552
	V_m_	0.029	1.030	0.965	1.100	0.379
	V_s_	0.003	1.003	0.958	1.051	0.891
	V_d_	0.104	1.110	1.016	1.211	0.020*
	AI	0.007	1.007	0.987	1.028	0.496
Sleepiness						
	ESS	0.085	1.089	0.985	1.203	0.096
	AIS	0.295	1.344	1.181	1.528	<0.001**
Psychological scale						
	SAS	0.196	1.216	1.096	1.349	<0.001**
POMS						
	tension anxiety	0.166	1.181	1.084	1.287	<0.001**
	depression	0.070	1.073	1.027	1.121	0.002**
	hostility	0.095	1.100	1.042	1.161	0.001**
	vigor	-0.077	0.926	0.879	0.976	0.004**
	fatigue	0.208	1.232	1.118	1.357	<0.001**
	confusion	0.216	1.241	1.099	1.402	<0.001**
Mental work capacity						
	simple reaction time	0.005	0.984	0.900	1.131	0.336
	attention span					
	digit symbols	0.001	1.001	0.978	1.024	0.938
	digit span	0	1.000	0.932	1.072	0.993
	pursuit aiming	-0.008	0.992	0.985	1.000	0.050*
Physical work capacity						
	PWC170	0	1.000	0.999	1.001	0.831
	FSAS	0.058	1.060	1.022	1.099	0.002**

*: p value is 0.05 or less;

**: p value is 0.01 or less.

Based on eigenvalue decomposition of the 16 original dimensions, 5 significant principal components (*i*.*e*., with an eigenvalue >1) were identified. These components explained 71.3% of the variance in the data. The first principal component, accounting for 30.7% of the variance, was dominated by the SAS, ESS, AIS and FSAS scores and the POMS. The variables that were predominant in the second component included HR; SpO_2_; and the SAS, ESS, AIS and FSAS scores. The principal components explaining the greatest variance in the data are presented in Table 4.

**Table 4:** Principal component analysis for AMS.

		PC1*(30.7%)	PC2* (14.5%)	PC3 (9.9%)	PC4 (8.4%)	PC5 (7.8%)
Demographic data						
	age	-0.129	-0.025	0.830	0.133	0.125
	BMI	0.009	0.018	0.798	-0.155	0.106
Physiological measurements						
	HR	-0.043	-0.514	-0.024	-0.532	0.395
	SpO_2_	-0.177	0.372	0.167	0.491	-0.547
	V_d_	0.035	-0.194	-0.363	0.480	0.349
Sleepiness						
	ESS	0.357	0.558	-0.047	0.156	0.287
	AIS	0.519	0.475	-0.027	-0.333	0.238
Psychological scale						
	SAS	0.590	0.578	0.023	0.096	0.282
POMS						
	tension anxiety	0.901	-0.235	0.091	-0.076	-0.175
	depression	0.902	-0.261	0.063	-0.011	-0.175
	hostility	0.872	-0.353	0.088	0.109	-0.108
	vigor	-0.188	-0.462	-0.029	0.405	0.366
	fatigue	0.908	-0.172	0.053	0.057	-0.045
	confusion	0.775	-0.317	-0.076	0.220	-0.020
Mental work capacity						
	pursuit aiming	-0.034	-0.071	0.261	0.415	0.435
Physical work capacity						
	FSAS	0.511	0.642	-0.083	-0.043	0.156

PC: Principal component.

*: p value is 0.05 or less.

We performed the adjusted regression analysis using 16 variables to identify independent risk factors for AMS. This analysis revealed that an elevated HR (odds ratio (OR): 1.047; p = 0.034) and V_d_ (OR: 1.173; p = 0.014), older age (OR: 1.279; p = 0.007), an increased AIS score (OR: 1.267; p = 0.030), decreased vigor (OR: 0.916; p = 0.015) and a decreased pursuit aiming capacity (OR: 0.988; p = 0.035) were independent risk factors for AMS (Table 5).

**Table 5:** Adjusted logistic regression.

Risk factor	β coefficient	OR	(95% CI)		p value
			Lower	Upper	
Age	0.246	1.279	1.070	1.528	0.007**
HR	0.046	1.047	1.003	1.093	0.034*
V_d_	0.159	1.173	1.032	1.332	0.014*
AIS	0.237	1.267	1.023	1.570	0.030*
Vigor	-0.088	0.916	0.854	0.983	0.015*
Pursuit aiming	-0.012	0.988	0.977	0.999	0.035*

CI: Confidence interval.

*: p value is 0.05 or less;

**: p value is 0.01 or less.

## Discussion

Our study described the clinical characteristics of patients with AMS and identified principal components associated with and risk factors for AMS using demographic data, routine physiological measurements, psychological scales and measurements of both sleep quality and mental and physical work capacity.

### Demographics and AMS

Age was found to be significantly associated with the AMS score. These results are partially in agreement with the results of previous studies that identified significant differences in the incidence of AMS between young and older subjects; these differences may be caused by associations between insomnia, headache and age [13]. However, other studies have shown that age is not an independent risk factor for AMS.

### The physiological characteristics of patients experiencing AMS

Regarding the systemic hemodynamics, only HR was significantly higher in the AMS+ group than in the AMS- group. Neither the SBP nor the DBP of the subjects was significantly associated with AMS. Despite the fact that the SpO_2_ of the patients who experienced AMS was not significantly different from that of the subjects without AMS, it was strongly negatively correlated with AMS scores, which has been previously reported [2, 8]. However, although it was previously reported that AMS is not related to CBF, as reflected by flow in the middle cerebral artery [14], individuals with AMS exhibited greater posterior cerebral circulation in the present study, as indicated by a higher V_d_ value, which is in agreement with previous reports [6, 15]. The altered hemodynamics in the AMS+ individuals may have been caused by activation of the sympathetic nervous system and alterations in the production of vasoconstrictors and vasodilators. This may also be attributed to the association between headache and the cerebral hemodynamics which has been indicated previously [16].

Regarding the echocardiographic parameters, only the PA of the subjects was positively correlated with their AMS score, which may have been due to the contraction of the pulmonary artery and which indicates that high-altitude pulmonary edema and AMS share mechanisms of action [17, 18]. Although it has been suggested that the SV increases immediately after exposure to acute hypoxia, cardiac function indexes were not closely related to the incidence of AMS in the present study, which may have been due to the increased HR and blood pressure [19].

### The role of psychological factors in patients experiencing AMS

The relationship between AMS and psychological factors has not been widely studied. We used psychological scales such as the SAS and characterized the mood of the subjects to examine this relationship. New-onset anxiety disorders have been reported at high altitudes, which may be related to AMS, as indicated by our and others’ studies [20, 21]. In the current study, the AMS patients exhibited greater anxiety than the individuals without AMS, as indicated by the differences in SAS scores between the AMS+ and the AMS- groups and the positive relationship between the SAS and the AMS scores. These phenomena may also have been caused by both a lack of knowledge of the effects of high altitude and anxiety about high altitude [22].

AMS is diagnosed based on subjectively self-reported symptoms; thus, psychological factors may partly explain the incidence of AMS. The association between AMS scores and anxiety specifically suggests that anxiety may contribute to the incidence of AMS, which is in agreement with the previous finding that anxiety critically contributes to AMS [23]. Though our previous study indicated that before high-altitude exposure, anxiety at sea level was associated with AMS [21], it cannot be excluded that anxiety may be caused and aggravated by AMS. Anxiety further results in somatization symptoms, such as headache and gastrointestinal symptoms, in AMS. The association between anxiety and AMS may be ascribed to the association between headache and the SAS score or to the psychological effects of the symptoms of AMS [24–27].

### The incidence of AMS is closely associated with the emotional state

It has been indicated that high-altitude exposure impairs mood and cognitive functions [28]. In the current study, all of the emotional parameters on the POMS questionnaire were highly correlated with the incidence of AMS, which is partly consistent with previous studies [23, 28, 29]. Specifically, tension anxiety was significantly higher among the patients who experienced AMS. Furthermore, anxiety was positively correlated with the AMS score of the participants. Thus, as suggested by the univariate logistic regression, anxiety may be a critical risk factor for AMS. Depression was also significantly more common among the subjects who experienced AMS, and a positive relationship between AMS and depression scores was observed, which may have been due to the association between depression and headache, and particularly high-altitude headache [24, 25, 30, 31]. In agreement with the results regarding anxiety and depression, AMS was characterized by higher levels of hostility and confusion. In contrast, the AMS patients did not exhibit more vigor, as indicated by the associations between the AIS score, fatigue and the AMS score; this finding may have been due to the fact that fatigue was assessed in the diagnosis of AMS. In addition, the POMS was associated with the likelihood of developing AMS, which warrants further study focusing on the mechanisms (including studies of the serological indexes, such as serotonin, involved in mood and emotions).

### Physiological work and mental exertion contribute to the development of AMS

Physiological labor capability reflects the cardiorespiratory functions of a subject, whereas mental labor capabilities represent the cognitive capacities. In the present study, although the cardiorespiratory functions of the subjects were altered significantly following exposure to high altitude, the PWC170 was similar in the subjects in the two groups. These findings may be explained by compensatory mechanisms undertaken by the cardiorespiratory system, which may have improved the PWC170 [9, 23]. However, as with fatigue, the FSAS score was closely related to the occurrence of AMS.

The cognitive capacity of each subject was measured using many variables. It has been reported that several cognitive capacities are impaired after high-altitude exposure, contributing significantly to AMS [32, 33]. The pursuit aiming score, which reflects movement stability, was significantly lower in the AMS+ group in the present study, indicating that the ability to pursue specific aims was impaired in individuals who developed AMS. The impaired cognitive functions of the patients may have been caused by acute hypobaric hypoxic stress and the consequent change in the CBF and oxygen supply.

### Principal component analysis and risk factors for AMS

We identified five principal components of AMS that explained 71.3% of the variance. Each principal component encompassed different aspects of AMS. Principal components 1 and 2 included many variables related to the POMS or emotions, whereas the other three principal components included physiological variables. The first component was dominated by fatigue, anxiety, depression, hostility and confusion. In contrast, the second principal component associated with AMS, which accounted for 14.5% of the variance in the AMS score, was largely dominated by sleepiness and the FSAS and SAS scores.

An elevated HR, older age, a greater AIS score, a higher V_d_, a lack of vigor and decreased pursuit aiming capacity were identified as independent risk factors for AMS. Several of these risk factors had previously been identified, whereas others represent novel findings [2, 3, 8, 34, 35]. In general, principal component analysis is largely underused when analyzing clinical data sets [36, 37]. In the current study, this type of analysis was used to reduce the dimensions of the variables and to screen risk factors for AMS in combination with logistic regressions, which may provide valuable directions and focuses for future research.

### Limitations

The subjects in our study were all young Chinese men, which could perhaps have generated bias due to age or gender; this aspect should be improved in our future studies. Additionally, the predictive roles of the risk factors and relevant mechanisms warrant further investigation. However, there are still many other risk factors for AMS that were not included in our study, such as pulmonary function, which should be investigated in the future. Another limitation is that the baseline of many parameters was not measured at 500 m due to the large sample size and our limited number of doctors; thus, only the cross-sectional characteristics at 3700 m were characterized in the present study. Therefore, to attain accurate causality between AMS and anxiety, more cohort or follow-up studies are needed.

## Conclusions

The likelihood of experiencing AMS was closely associated with the subject’s mood, including anxiety, depression, hostility, vigor, fatigue and confusion, in addition to HR and V_d_. Moreover, older individuals and individuals with an elevated HR, an increased posterior cerebral circulation velocity, an increased AIS score, a lack of vigor or a partially impaired mental work capacity were at greater risk of experiencing AMS.

## Supporting Information

S1 File **Document A**, Lake Louise self-scoring system. **Document B**, Self-Rating Anxiety Scale questionnaires. **Document C**, Profile of mood states questionnaire. **Document D**, Epworth Sleepiness Scale. **Document E**, Athens Insomnia Scale. **Document F**, Cognitive function tests. **Document G**, Fatigue Self-Assessment Scale. **Document H**, Measurements of PWC170.
(RAR)

## Abbreviations

AMS: acute mountain sickness
AIS: Athens Insomnia Scale
BMI: body mass index
EF: ejection fraction
ESS: Epworth Sleepiness Scale
FSAS: Fatigue Self-Assessment Scale
HR: heart rate
LA: end-diastolic internal diameter of the left atrium
LLS: Lake Louise Score (self-assessment scoring system)
LV: end-diastolic internal diameter of the left ventricle
PA: end-diastolic internal diameter of the pulmonary artery
POMS: profile of mood states
PWC170: physical working capacity when the HR is 170 beats/min
RA: end-diastolic internal diameter of the right atrium
RV: end-diastolic internal diameter of the right ventricle
SAS: Self-Rating Anxiety Scale
SpO_2_: oxygen saturation
SV: stroke volume
